# Predictors of Hospitalization for Manic Episode in Alzheimer’s Dementia: Inputs From an Inpatient Case-Control Study

**DOI:** 10.7759/cureus.17333

**Published:** 2021-08-20

**Authors:** Varsha Nandwana, Jaskaranpreet Kaur, Ripudaman Singh, Sanobar Jaka, Gagan Kaur, Era Rawal, Keerthika Mathialagan, Ozge C Amuk Williams

**Affiliations:** 1 Medicine, Lady Hardinge Medical College, Delhi, IND; 2 Medicine, Dayanand Medical College & Hospital, Ludhiana, IND; 3 Internal Medicine, Jawaharlal Nehru Medical College, Wardha, IND; 4 School of Global Public Health, New York University, New York, USA; 5 Medicine and Surgery, Sri Guru Ram Das Institute of Medical Sciences & Research, Amritsar, IND; 6 Medicine/Cardiology, Norvic International Hospital, Kathmandu, NPL; 7 Psychiatry, Sree Balaji Medical College and Hospital, Chennai, IND; 8 Psychiatry, Griffin Memorial Hospital, Norman, USA

**Keywords:** alzheimer’s dementia, progressive dementia, manic episodes, hospitalization outcomes, demographic characteristics, risk factors

## Abstract

Objectives

The correlates of manic episodes in dementia have not been systematically studied. The primary goal of our study is to compare the sociodemographic characteristics and psychiatric comorbidities in Alzheimer’s dementia (AD) inpatients with manic episodes versus without manic episodes, and to evaluate the demographic predictors and risk factors for manic episodes in AD inpatients.

Methods

We conducted a case-control study using the Nationwide Inpatient Sample of 34,285 AD patients (age ≥60 years). Subsequently, the cases i.e., AD inpatients with a manic episode (N = 1,035) and the controls (without a manic episode, N = 1,035), were extracted using propensity-score matching based on age. The cases did not have a past psychiatric history of bipolar disorders. We used the logistic regression model to evaluate the odds ratio (OR) of association between pre-existing psychiatric comorbidities and manic episodes and evaluate the demographic predictors of manic episodes in AD inpatients.

Results

A higher proportion of AD inpatients with manic episodes were females (63.8%), whites (85.2%), and from low-income families below the 50^th^ percentile (63%). Females were more likely to be hospitalized for manic episodes (OR 1.33; 95% CI 1.09-1.64) than males. AD inpatients with manic episodes had a higher risk of presenting with suicidal behaviors (OR 1.88; 95% CI 1.23-2.86). A significantly higher proportion of AD inpatients with manic episodes had comorbid tobacco use (5.3% vs. 3.4%) and cannabis use (1.4% vs. 0%) compared to those without manic episodes.

Conclusion

Females with AD had a greater risk of being hospitalized for manic episodes. These patients have an 88% higher risk of suicidal behaviors during the manic presentation and have comorbid tobacco and cannabis use. Early diagnosis and management of manic episodes in at-risk AD patients are important to improve the quality of life (QoL) and outcomes.

## Introduction

Alzheimer’s dementia (AD) is a progressive neurodegenerative disease characterized by memory loss, cognitive deficits, and behavioral changes. Up until 2020, more than 5.5 million Americans, including an estimated 5.3 million people aged 65 years and older, had AD dementia, and two-thirds of them were women. AD is the fifth leading cause of death in the United States (US) for women and the eighth leading cause of death among men [1]. By 2021, of the 6.2 million people aged 65 and older with AD in the US, 3.8 million were women, and 2.4 million were men, and the incidence of AD and other dementias is projected to double by 2050 [2]. Older black and Hispanics are disproportionately more likely to have AD or other dementias than older white Americans. Data from the Chicago Health and Aging Project (CHAP) population-based study of chronic health conditions indicate that 18.6% of blacks and 14% of Hispanics age 65 and older have AD compared with 10% of White older adults [3].

According to past studies, adults with bipolar disorder suffer from cognitive impairment with difficulties in attentional processing and executive function [4]. Several neurobiological and pathophysiological pathways are similar between AD and bipolar disorder, including hyper inflammation, involvement of the kynurenine pathway, increased oxidative stress, and reduced neurotrophic support [5,6]. Some studies have shown that patients with a history of bipolar disorder had an increased risk of developing dementia later in life. The prevalence of mania is notably higher in patients with dementia, 1% in the general population compared to 3.8% in dementia patients [7]. This risk is compounded by pre-existing risk factors, including genetic susceptibility, substance abuse, poor lifestyle, and medical comorbidities [5].

A history of psychiatric illness is one of the most significant factors associated with an increased risk of dementia, and depressive episodes being the most common psychiatric diagnosis [8]. A case-control study done on the Danish population found out that patients with dementia were more likely to be admitted for mania. However, this study did not establish a temporal relationship between the two illnesses [9].

It is crucial to explore the association of manic episodes in AD patients; since the management of behavioral and mood symptoms is one of the most challenging aspects of AD. On average, the rate of dementia increases by 6% with every episode leading to admission for patients with bipolar disorder [10]. It is clinically important to identify the risk factors for manic episodes in patients with AD. Early detection can improve the quality of life (QoL) among AD patients and also help in reducing the cost burden on the healthcare system.

While past research has established the association between dementia and major depressive disorder, limited studies have explored the relationship between dementia and bipolar disorder and/ manic episode. The goals of our study are to discern the differences in sociodemographic characteristics, psychiatric comorbidities, and substance use disorders (SUD) in AD patients hospitalized for manic episodes versus those with manic episodes. Next, we will measure the demographic predictors and risk factors for manic episodes in inpatients with AD and no previous history of bipolar disorder.

## Materials and methods

Study sample

We conducted a cross-sectional study using the Nationwide Inpatient Sample (NIS) from 2012 to 2014. The NIS is the largest inpatient database collected from 4,411 non-federal hospitals across 44 states in the US [11].

Our initial sample included 34,285 patients (age ≥60 years) with Alzheimer’s dementia (AD) and was grouped by a primary discharge diagnosis of a manic episode, unspecified. The diagnosis of a manic episode, unspecified, is given to patients who do not completely meet the diagnostic criteria for bipolar disorders and/ have no past psychiatric history of bipolar disorders. The prevalence of manic episodes in AD inpatients was 3%. We used propensity score matching based on age at admission to extract the cases i.e., AD inpatients with manic episodes (N = 1,035) and the controls i.e. AD inpatients without a manic episode (N = 1,035).

Variables

The demographic variables included in this study were age at admission, sex, race, and median household income. Psychiatric comorbidities were defined by various co-diagnoses in the patient’s records. We included suicidal behaviors, anxiety disorders, post-traumatic stress disorders (PTSD) and psychotic disorders, and alcohol use disorders and substance use disorders (tobacco, cannabis, and stimulants) as the variables of interest [12].

Statistical analysis

We compared the distributions of demographic characteristics and psychiatric comorbidities in AD inpatients without versus with manic episodes by performing descriptive statistics and Pearson’s chi-square test. Next, we used the logistic regression model to evaluate the odds ratio (OR) of association between psychiatric comorbidities and manic episodes and evaluate the demographic predictors of manic episodes in AD inpatients. In this regression model, we only included the variables which were statistically significant in the Pearson’s chi-square test. All analyses were conducted using the Statistical Package for the Social Sciences (SPSS) version 26.0 (IBM Corp., Armonk, NY) and statistical significance was set at a two-sided P-value <0.05.

Ethical approval

The NIS is publicly available de-identified data with the protection of patients, physicians, and hospital-related information; henceforward, we were not required to take institutional review board permission for our study [11].

## Results

Our study population included 34,285 patients with AD. Among the inpatients admitted for a manic episode, the majority were females (63.8%) and whites (85.2%). The prevalence rate of manic episodes among inpatients also differed by socioeconomic status. Inpatients from low-income families below the 50^th^ percentile for median income had a higher prevalence of manic episodes (63%).

Anxiety disorder was the most prevalent comorbidity in the study sample and there existed a non-significant difference in the prevalence between the groups. AD inpatients with manic episodes had a significantly higher prevalence of suicidal behaviors (7.7% vs. 4.8%) compared to those without manic episodes. A significantly higher proportion of AD inpatients with manic episodes had comorbid tobacco use (5.3% vs. 3.4%) and cannabis use disorder (1.4% vs. 0%) as shown in Table 1.

**Table 1:** Distribution of demographics and comorbidities in Alzheimer’s dementia inpatients by manic episodes SD: standard deviation.

Variable	Manic episode		P-value
	No	Yes	
Mean age (SD), in years	75.6 (7.7)	75.6 (7.7)	1.000
Sex, in %			
Male	43.5	36.2	0.001
Female	56.5	63.8	
Race, in %			
White	89.2	85.2	<0.001
Black	3.9	6.1	
Hispanic	3.9	7.1	
Others	2.9	1.5	
Median household income, in %			
Below 50^th^ percentile	29.9	63.0	<0.001
Above 50^th^ percentile	70.1	37.0	
Psychiatric comorbidities, in %			
Suicidal behaviors	4.8	7.7	0.007
Anxiety disorders	23.2	20.3	0.110
Post-traumatic stress disorder	3.4	1.4	0.004
Psychotic disorders	18.8	0.5	<0.001
Substance use disorders, in %			
Alcohol	4.8	5.3	0.616
Tobacco	3.4	5.3	0.031
Cannabis	0	1.4	<0.001
Stimulants	-	-	-

Females with AD are more likely (OR: 1.33; 95% CI 1.09-1.64) to be hospitalized for manic episodes than males. When compared to whites, blacks had an increased odds for manic episodes, but the results were not statistically significant (OR: 1.34; 95% CI 0.85-2.09). Other races had a non-significant negative association with manic episodes. Among socio-economic classes, the higher-income percentile was at a lower risk for manic episodes (OR: 0.51; 95% CI 0.46-0.57).

AD inpatients with manic episodes were at an increased risk of presenting with suicidal behaviors (OR: 1.88; 95% CI 1.23-2.86) compared to those without manic episodes. Also, patients with comorbid tobacco use disorders are at higher odds for manic episodes (OR: 1.86; 95% CI 1.03-3.38) as shown in Table 2.

**Table 2:** Predictors of a manic episode in Alzheimer’s dementia inpatients

Variable	Logistic regression model		P-value
	Odds ratio	95% confidence interval	
Sex			
Male	Reference		
Female	1.33	1.09-1.64	0.001
Race, in %			
White	Reference		
Black	1.34	0.85-2.09	0.212
Hispanic	0.94	0.59-1.49	0.780
Others	0.52	0.24-1.13	0.096
Median household income, in %			
Below 50^th^ percentile	Reference		
Above 50^th^ percentile	0.51	0.46-0.57	<0.001
Psychiatric comorbidities, in %			
Suicidal behaviors	1.88	1.23-2.86	0.003
Post-traumatic stress disorder	0.38	0.19-0.77	0.007
Psychotic disorders	0.03	0.01-0.06	<0.001
Substance use disorders, in %			
Tobacco	1.86	1.03-3.38	0.041
Cannabis	-	-	0.999

## Discussion

In this cross-sectional inpatient study of 34,285 patients with AD and no past diagnosis bipolar disorders, 3% had manic episodes, which are comparable to the 6% overall prevalence of late-life mania [13]. There is very limited research on mania in the elderly population since its symptoms overlap with many other organic and chronic diseases and are often erroneously interpreted as delirium [14]. It is imperative to understand the association of manic episodes in AD patients to draw meaningful epidemiologic and prognostic factors for acute and chronic care management of the elderly.

The patient with AD in our study had a mean age of 75.6 and constituted majorly by females (60.2%) and whites (87.2%), which is consistent with the demographic distribution from the US Census 2016 report [15]. Females are more likely to suffer from AD and are also more likely to experience manic episodes following AD possibly due to an overall higher probability of diagnosing psychiatric illness among females [2,16]. High-income percentile AD patients were at lower odds of getting hospitalized due to manic episodes. This finding could potentially reflect the inverse relationship between socioeconomic status and dementia diagnosis [17]. The population with higher education and higher income class is hypothesized to have built “cognitive reserve” and could be a protective factor for AD [18].

Race was not a statistically significant predictor of mania in AD, although significantly limited by the health disparities among minority communities and access to quality primary and geriatric care. Prior psychotic episodes, tobacco use, and a low household income were strongly associated with manic episodes in AD. Bipolar disorder has the highest prevalence of SUD than any other psychiatric illness [19]. Past literature exploring the association between cannabis use and exacerbation of manic episodes revealed a three-fold increased risk of manic episodes [20]. In our study, we found a significantly higher prevalence of comorbid tobacco use and cannabis use among AD patients presenting with manic episodes. Additionally, we found a statistically significant association between AD inpatients presenting with suicidal behaviors than non-manic episodes. AD is a known risk factor for suicidal behavior in elderly patients even years after the diagnosis [21].

It is important to further study the prevalence and the underlying risk factors for late-onset bipolar illness as it further potentiates the risk of developing dementia. Azorin et al. mentioned the term bipolar type VI i.e. the bipolarity in the context of dementia-like processes, which may help the physicians to orientate both diagnosis and treatment decisions [22].

Our study results should be considered with some limitations. Potential diagnosis of a manic episode may be masked with delirium in patients with dementia due to the overlapping nature of the presentation. As a limitation of a retrospective, observational study, we could not establish a causal relationship between AD and manic episodes. These findings from the dataset may underreport other comorbidities which are pre-existing conditions/diagnoses in the patient records. The diagnoses for comorbid substance use in remission were excluded from our study but we cannot confirm the continuous/episodic use of the substance and its relationship with manic episodes. The strength of our study is that we used the NIS, which provides a population-based dataset with national representation. The results are sufficiently generalizable to the inpatient population, and there is a low risk of reporting bias since the data codes are independently reported by an individual practitioner.

## Conclusions

Manic episodes in AD, albeit relatively rare occurrence, is a significant deterrent of QoL among elderly patients. Females with AD were at higher risk of hospitalizing for manic episodes. Meanwhile, a higher socioeconomic status was a relatively protective factor against developing mania among AD inpatients. These at-risk AD patients with manic episodes have an 88% higher risk of suicidal behaviors compared to patients without manic episode presentation. Also, comorbid tobacco use and cannabis use increases the likelihood of manic episode-related hospitalization. Early diagnosis and management of manic episodes in at-risk AD patients are important to improve the QoL and outcomes.
